# Atypical response to erlotinib in a patient with metastatic lung adenocarcinoma: a case report

**DOI:** 10.1186/1752-1947-8-335

**Published:** 2014-10-09

**Authors:** Linda Sakhri, Elodie Meynet, Léonie Ferrer, Augustin Pirvu, Gilbert Ferretti, Denis Moro-Sibilot

**Affiliations:** 1 Pôle thorax et vaisseaux, Unité d’oncologie thoracique, CHU Grenoble, BP217, 38043 Grenoble cedex 9, France; 2 Service de chirurgie thoracique, vasculaire et endocrinienne, CHU Grenoble, BP217, 38043 Grenoble cedex 9, France; 3 Service de radiologie, CHU Grenoble, 38043 Grenoble cedex 9, France

**Keywords:** Bilateral pneumothorax, EGFR mutation, Erlotinib, Pulmonary adenocarcinoma, Pulmonary cysts, Tyrosine kinase inhibitors

## Abstract

**Introduction:**

Tyrosine kinase inhibitors are widely prescribed in thoracic oncology and have excellent responses as a first-line treatment for locally advanced or metastatic lung cancer with epidermal growth factor receptor mutations. The side effects of tyrosine kinase inhibitors are mostly gastrointestinal and dermatological, and are usually resolved after symptomatic treatment. However, new complications have now arisen due to increased use of these drugs. Here we report a side effect of erlotinib that has not been described previously: that is, metastatic lung tumor nodules were transformed into cysts, which ruptured the pleura and were responsible for bilateral life-threatening pneumothorax.

**Case presentation:**

We report the case of a 35-year-old Caucasian woman with metastatic adenocarcinoma and a deletion in epidermal growth factor receptor exon 19 (del E746-A750). She was treated with erlotinib for metastatic lung adenocarcinoma.

Treatment with erlotinib resulted in the replacement of pulmonary tumor nodules with air-containing cysts. These cysts ruptured in the pleura causing a life-threatening bilateral pneumothorax.

To the best of our knowledge, this tumor–cystic response after erlotinib therapy has not been previously described.

**Conclusions:**

Tyrosine kinase inhibitors are widely prescribed in thoracic oncology, and managing toxicities must be optimal in order to improve adherence. Transformation of pulmonary nodules into cysts must be known and clinicians should be aware of this potential complication, which can lead to life-threatening pneumothorax.

## Introduction

Targeted therapies in thoracic oncology are starting to compete with ‘classic’ chemotherapies due to their better clinical tolerance and easier mode of administration. However, we are now faced with a new complication because of their increased usage. Tyrosine kinase inhibitors (TKIs) are now widely prescribed in thoracic oncology: thus, managing any toxicity should be optimal to improve adherence. Here we report a case in which erlotinib caused transformation of pulmonary nodules into cysts; this has not been described previously in the literature.

## Case presentation

**Figure 1:**
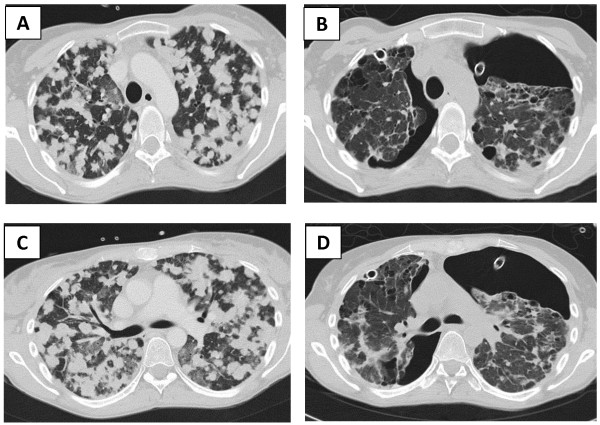
**Thoracic computed tomography scan. (A) (C)** Initial thoracic computed tomography scan showing multiple pulmonary metastases. **(B) (D)** Thoracic computed tomography scan after 2 months of treatment with erlotinib, showing a partial thoracic response with the appearance of pulmonary cysts and a partially drained bilateral pneumothorax.

**Figure 2:**
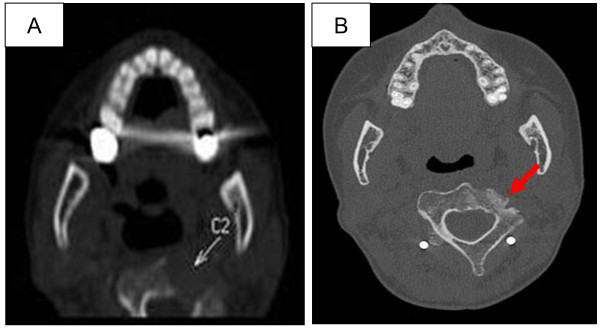
**Computed tomography scan of the neck. (A)** revealing C2 osteolysis as indicated by the arrow. **(B)** showing osteocondensation (as indicated by the arrow) in the location of the initial lysis of the C2 vertebrae after 2 months of treatment with erlotinib.

A 35-year-old Caucasian woman and former cigarette smoker presented with dyspnea and neck pain. A computed tomography (CT) scan revealed bilateral round opacities in both lungs (Figure 1A, C), hepatic metastases, osteolysis of her second cervical vertebra (C2) (Figure 2A), and two cerebral metastases. A percutaneous biopsy of the hepatic metastasis showed lung adenocarcinoma with a deletion in the epidermal growth factor receptor exon 19 (del E746-A750).Treatment with erlotinib was started. After 2 months she was hospitalized in our intensive care unit for an acute respiratory distress syndrome, secondary to a bilateral and spontaneous pneumothorax, which required placement of a chest tube. A thoracic CT scan revealed the replacement of diffuse parenchymatous nodules by cystic lesions (Figure 1B, D). Because of prolonged air leakage but also a good response to oncological treatment, a two-stage thoracoscopic bilateral talc poudrage was performed. Her postoperative course was uneventful and the drains were removed at 3 weeks after surgery.

The cancer progressed within the next 9 months. A second-line treatment of cisplatin, pemetrexed, and bevacizumab followed by a maintenance therapy of bevacizumab, was given. After 6 months of chemotherapy, the tumor had progressed and erlotinib was reintroduced. This therapy was associated with a cerebral infarction in the context of paraneoplastic syndrome. The patient died 18 months later from disease progression.

## Discussion

In this patient, erlotinib gave an excellent initial response; there was complete disappearance of the cerebral metastases, and the osteocondensation of the C2 vertebrae (Figure 2B). However, lysis of the subpleural metastases (Figure 1B, C) could explain the occurrence of the cystic lesions and pneumothorax.

We have recently reported a case in which there was transformation of pulmonary nodules into pulmonary cysts in a 40-year-old woman who was a non-cigarette smoker with multi-metastatic pulmonary adenocarcinoma, an *EML4-ALK* gene rearrangement, and who was successfully treated with crizotinib [1].

The phenomenon of cyst formation is caused by parenchymal necrosis as a result of targeted therapy, but which also increases the risk of a pneumothorax. Tumor necrosis has previously been described in connection with the lysis of some chemosensitive tumors, such as germinal tumors, lymphomas, and sarcomas [2,3], with a 1% risk of a pneumothorax occurring within 2 to 7 days following the start of chemotherapy [4].

Transformation of tumor lesions into cysts is also a difficulty in Response Evaluation Criteria in Solid Tumours assessments. The solid tumors initially retain the same diameter as the newly formed cysts.

TKIs are prescribed as a first-line therapy in the context of sensitive mutations, and produce a response rate of 50 to 90% and progression-free survival times of 9.7 to 13.1 months; this is compared to 4.6 to 5.2 months in patients treated with a platinum-based chemotherapy [5].

Here we report the unusual response of the lung after erlotinib therapy, in which the lung nodules were transformed into cysts via a mechanism that was probably associated with necrosis.

## Conclusions

The association between lysis of a lung tumor and its replacement by a cyst seemed to be caused by erlotinib treatment: thus, this should be borne in mind, as the rupture of a cyst in the pleura can result in a life-threatening pneumothorax.

## Consent

Written informed consent was obtained from the patient's next of kin for publication of this case report and accompanying images. A copy of the written consent is available for review by the Editor-in-Chief of this journal.

## Competing interests

DMS declares that he is on the advisory board and consults for Roche, Astra Zeneca, Pfizer and Lilly Laboratories. GF declares that he received honorarium and travel expenses to speak to professional groups, travel accommodations and meeting expenses (Roche and Guerbet laboratories), and payment for lectures including service on speakers bureaus (Lilly, Boehringer, Actelion, Astra Zeneca, Roche). All other authors have no conflicts to declare.

## Authors’ contributions

LS wrote the manuscript and helped manage the patient while in the hospital. EM helped manage the patient while in the hospital and was a major contributor in writing the manuscript. LF helped manage the patient while in the hospital and was a major contributor in writing the manuscript. AP was the surgeon of the patient and a major contributor in writing the manuscript. GF interpreted the radiologic data and was a major contributor in writing the manuscript. DMS helped manage the patient while in the hospital and was a major contributor in writing the manuscript. All authors read and approved the final manuscript
